# Toward the Restoration of Hand Use to a Paralyzed Monkey: Brain-Controlled Functional Electrical Stimulation of Forearm Muscles

**DOI:** 10.1371/journal.pone.0005924

**Published:** 2009-06-15

**Authors:** Eric A. Pohlmeyer, Emily R. Oby, Eric J. Perreault, Sara A. Solla, Kevin L. Kilgore, Robert F. Kirsch, Lee E. Miller

**Affiliations:** 1 Department of Physiology, Feinberg School of Medicine, Northwestern University, Chicago, Illinois, United States of America; 2 Department of Biomedical Engineering, Northwestern University, Evanston, Illinois, United States of America; 3 Department of Physical Medicine and Rehabilitation, Feinberg School of Medicine, Northwestern University, Chicago, Illinois, United States of America; 4 Department of Physics and Astronomy, Northwestern University, Evanston, Illinois, United States of America; 5 Department of Biomedical Engineering, Case Western Reserve University, Cleveland, Ohio, United States of America; 6 MetroHealth Medical Center, Cleveland, Ohio, United States of America; 7 Louis Stokes Cleveland Veterans Affairs Medical Center, Cleveland, Ohio, United States of America; The Research Center of Neurobiology - Neurophysiology of Marseille, France

## Abstract

Loss of hand use is considered by many spinal cord injury survivors to be the most devastating consequence of their injury. Functional electrical stimulation (FES) of forearm and hand muscles has been used to provide basic, voluntary hand grasp to hundreds of human patients. Current approaches typically grade pre-programmed patterns of muscle activation using simple control signals, such as those derived from residual movement or muscle activity. However, the use of such fixed stimulation patterns limits hand function to the few tasks programmed into the controller. In contrast, we are developing a system that uses neural signals recorded from a multi-electrode array implanted in the motor cortex; this system has the potential to provide independent control of multiple muscles over a broad range of functional tasks. Two monkeys were able to use this cortically controlled FES system to control the contraction of four forearm muscles despite temporary limb paralysis. The amount of wrist force the monkeys were able to produce in a one-dimensional force tracking task was significantly increased. Furthermore, the monkeys were able to control the magnitude and time course of the force with sufficient accuracy to track visually displayed force targets at speeds reduced by only one-third to one-half of normal. Although these results were achieved by controlling only four muscles, there is no fundamental reason why the same methods could not be scaled up to control a larger number of muscles. We believe these results provide an important proof of concept that brain-controlled FES prostheses could ultimately be of great benefit to paralyzed patients with injuries in the mid-cervical spinal cord.

## Introduction

Many spinal cord injury survivors report that recovery of hand use would be the most desirable function to regain [1]. To this end, functional electrical stimulation (FES) has been used to restore limited, but functionally important grasping to several hundred human spinal cord injured patients [2], [3], [4]. However, despite their success, the most advanced systems are restricted to several pre-programmed grasp patterns that are under proportional control through single degree of freedom sensors and mode switches actuated by residual voluntary movement or by muscle activity of the proximal limb, wrist or head [5]. There have also been efforts to use electroencephalographic (EEG) signals to trigger similar preprogrammed sequences [6], [7], [8]. Despite these advances, the goal of achieving truly dexterous manipulation of objects remains elusive.

By now, a number of groups have shown that multi-electrode recordings from the primary motor cortex (M1) can be used to predict kinematic features of desired movement [9], [10], [11], [12] and that these signals can be used for real-time control of movement kinematics [13], [14], [15], [16], [17], [18], [19]. However, there is abundant evidence that neurons in M1 carry information related to the dynamics of movement as well as kinematics [20], [21], [22], [23], [24], [25], [26]. We have previously shown that such signals can be used to predict the muscle activity (EMG) underlying complex reaching tasks [27]. Here we report an important proof of concept experiment using real-time EMG predictions to control electrical stimulation of several forearm muscles of monkey subjects. This brain-controlled FES restored limited, voluntary movement during temporarily paralysis of the arm. Two paralyzed monkey subjects roughly doubled their maximum voluntary wrist flexion force, and were able to grade the force with sufficient accuracy to match a cursor to targets at different force levels. We are currently working to refine this approach to allow voluntary control of more complex and varied hand movements. We anticipate that the approach could offer significant advantages to paralyzed patients with injuries in the mid-cervical spinal cord, and potentially even greater benefits to patients with high-cervical injuries resulting in paralysis of the entire upper limb.

## Results

### Voluntary Control of Paralyzed Muscles

**Figure 1:**
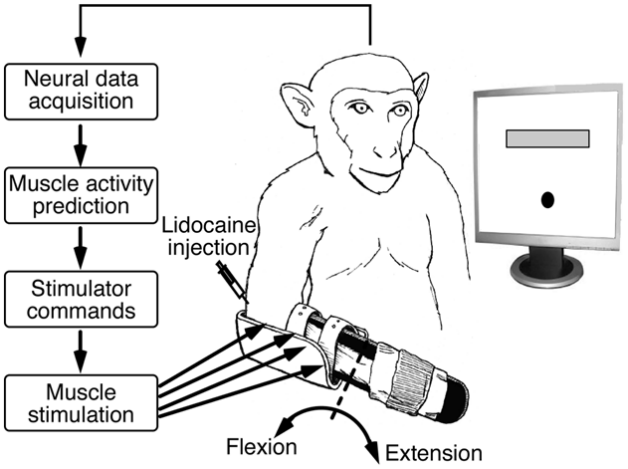
Direct brain control of functional electrical stimulation (FES) for wrist movement. A force-controlled cursor and a target were displayed on a computer monitor. Real-time predictions of desired muscle activation were generated from motor cortical activity and used to control the electrical stimulation of four muscles. Two monkeys could generate wrist force voluntarily despite the paralysis of wrist muscles by peripheral nerve blocks.

We performed a series of experiments with two *rhesus macaque* monkeys (monkeys A and T). Figure 1 illustrates the essential components of these experiments. Each monkey faced a video monitor that displayed a circular cursor and a rectangular target. The monkey controlled the position of the cursor by exerting flexion or extension forces at the wrist. Temporary paralysis was induced by pharmacological blocks of the median and ulnar nerves at the elbow, which affected the intrinsic hand muscles and extrinsic wrist and finger flexor muscles, while leaving the extensors intact.

We used recordings from a multi-electrode array chronically implanted in the primary motor cortex (M1) to generate real-time predictions of intended muscle activation. These predictions were used to control the intensity of stimulation to four forearm flexor muscles, thus providing a brain interface by which the monkey could voluntarily control its paralyzed muscles. We quantified the effectiveness of this control in terms of: 1) the increase in voluntary force generating capacity, 2) the similarity in the time course of the force under normal and FES conditions, and 3) the precision with which the force was controlled.

**Figure 2:**
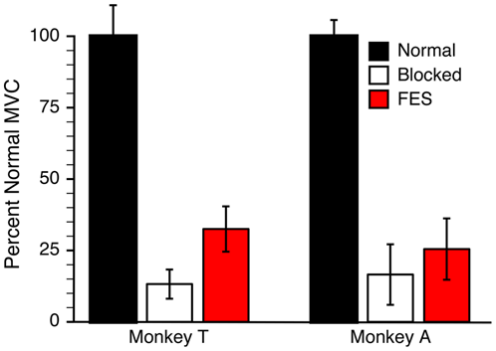
Mean +/− SD of the maximum wrist force generated under normal, nerve block, and FES conditions. Nerve blocks (white bars) resulted in greatly diminished wrist strength compared to normal (black bars), but both monkeys were able to generate greater force during the block when using brain-controlled FES (red bars).

The nerve block dramatically decreased the amount of wrist flexion force that the monkeys could generate voluntarily. Figure 2 summarizes this effectiveness, as well as the increase in force afforded by the brain-controlled FES. We estimated maximum voluntary contraction (MVC) under normal, blocked, and FES conditions by measuring the maximum force that the monkey could maintain for 0.5 seconds. This corresponded to the required target hold time during the behavioral task (see supplementary materials, “Methods S1”). For monkey T, MVC generated in the blocked state without FES (“Blocked MVC”) averaged 13% of normal across nine sessions. For monkey A, the average Blocked MVC was 17% of normal across four sessions. The difference in MVC between the normal and blocked states was highly significant for both monkeys (paired t-tests, p≪.001).

It seemed apparent by watching the monkeys that some of the remaining force in the blocked state resulted from the action of unblocked, proximal muscles that was inappropriately registered by the wrist force transducer. Unfortunately, quantifying the magnitude of this effect is difficult. The magnitude of EMG from the blocked muscles was very near the noise level; its reduction from the normal level was greater than the corresponding reduction in force (ANOVA, p≪0.001). For monkey T, the average ratio of the EMG between the normal and blocked MVC tests in nine sessions was only 1.5% for the major wrist and digit flexors. The results for monkey A (for which the nerve blocks were done without implanted canulae; see supplementary materials, “Methods S1”) were similar, with the exception of flexor carpi radialis (FCR). For monkey A, the average blocked EMG activity in flexor digitorum profundus (FDP), flexor digitorum superficialis (FDS), flexor carpi ulnaris (FCU), and palmaris longus (PaL) was only 3% of normal. In contrast, the remaining FCR EMG varied from 20 to 80% or normal, in part because there may have been significant electrical crosstalk from the nearby unblocked brachioradialis.

Even this small level of EMG might, nevertheless, have accounted for a disproportionate amount of the remaining force. However, if this had been a significant effect, the magnitude of the remaining force, which varied across sessions, should have been related to the magnitude of the remaining EMG. This was not the case for any of the muscles from either monkey (R^2^<0.08; p>0.24). This logic leads us to conclude that the remaining force in the blocked state was primarily due to unblocked muscles, and that the estimated MVC force in the blocked state was probably overestimated (see supplementary materials, “Methods S1”).

With the FES system active, MVC increased above the blocked level to 25% and 33% of normal for monkeys A and T, respectively. The differences between the Blocked and FES MVCs were significant (paired t-tests, p≪.03 monkey A; p = .001 monkey T). Unless otherwise noted, all subsequent force-related results are expressed relative to the Blocked MVC.

**Figure 3:**
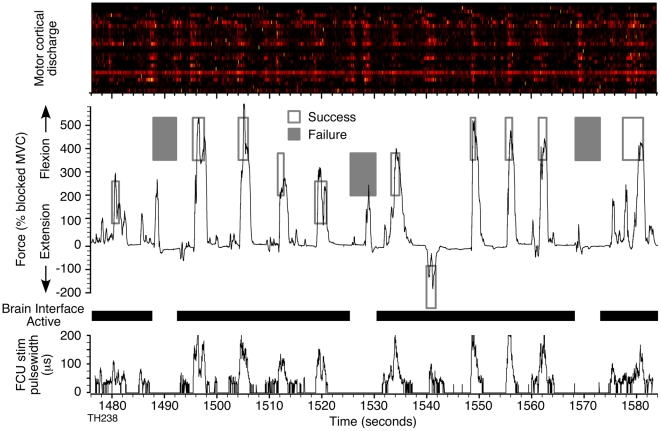
Brain-controlled FES command signal and resulting force. Uppermost panel shows the modulation of the 25 neurons used for control. The discharge of each neuron has been normalized to the peak rate that occurred within this segment of data. The FES-mediated force curve produced by monkey T during a continuous series of trials to different force targets (rectangles) is shown immediately below. Targets for successful trials are shown by open rectangles. Failed trials (filled rectangles) occurred only during random catch trials in which the brain interface and FES were not active (gaps in the heavy black bar). The bottom trace shows the FES pulse widths for wrist muscle flexor carpi ulnaris (FCU).

Beyond simply generating larger forces, the brain-controlled FES system allowed both monkeys to grade the amount of force they produced as would be necessary for a useful clinical application. Figure 3 shows a short time segment of the force generated by monkey T during an FES session in which four distinct wrist force targets (three flexion and one extension) were presented. A video clip of the performance in a similar session is included in the supplementary material (“Brain Controlled FES S1”). In this particular session, the monkey controlled the stimulus-driven activity of PaL, FDS, FCU, and FDP. The rectangles in Figure 3 indicate the upper and lower force limits of the targets and the timing of their presentation. The force had to be maintained within a target for 0.5 seconds for a trial to be a success (open rectangles). The 25 neurons used for control were clearly modulated during force generation. The individual patterns are difficult to discern at this time scale, but some variety across neurons and across trials can be appreciated. At the bottom of the figure are shown the pulse widths of the stimuli derived from this neural discharge that were used to activate the wrist flexor FCU.

In several experiments with monkey T, the FES system was intentionally turned off for 20% of randomly selected “catch” trials (shown in Figure 3 as gaps in the “Brain Interface Active” bar). None of the catch trials was successful in this particular example. Note however, that the monkey was clearly making an effort to generate force on each of these trials, based on the accompanying patterns of neural discharge. Note also that force peaks generated during catch trials were much narrower than those during FES trials, as the monkey was unable to generate even low level sustained force without the FES; it appeared as though the narrow force transients resulted primarily from inertial forces coupled to the hand as the proximal limb was accelerated. The average catch trial success rate was only 2% for targets at or above the Blocked MVC. These few successes occurred in a single session in which the lowest edge of a target had been placed at the edge of the Blocked MVC. By contrast, when the brain interface and stimulators were active, both monkeys were consistently able to reach force targets that were above their Blocked MVC. We completed a total of six sessions over a five-week period with monkey T. Each of these sessions consisted of approximately 125 trials with an average success rate of 81%. With monkey A, we completed two sessions with a 90% average success rate. Because of the additional complication of needing to inject lidocaine directly to the nerve, these sessions were shorter, averaging 80 trials in length. Average success rates during sessions without a block (normal conditions) were 91% (monkey T) and 99% (monkey A) for flexion targets. For extension targets, both monkeys successfully completed over 90% of the FES trials, essentially the same as their performance under normal (unblocked) conditions (monkey T: 89% and monkey A: 100%).

### Comparison of Normal and FES Force Control

**Figure 4:**
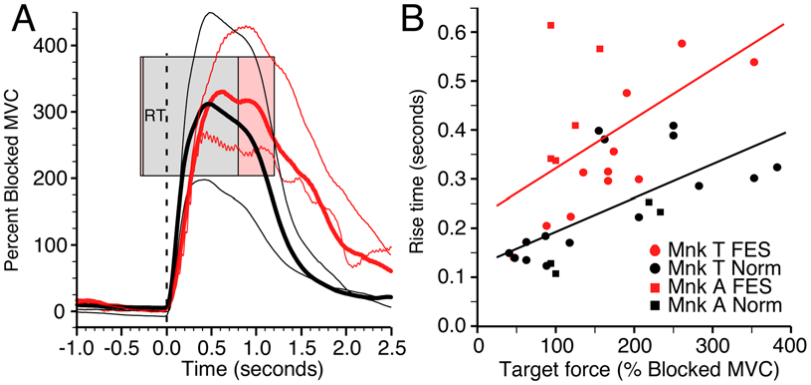
Time course of normal and FES-generated force. (A) Averaged force during FES (red) and normal (black) sessions with matched targets (monkey T). Pink and gray rectangles represent the top, bottom, and average duration of the targets in FES and normal conditions, respectively. Because the force traces are aligned to force onset (vertical dashed line), the left edge of the target (indicating the time of its appearance) is dependent on the reaction time. Note that the left edge of the gray rectangle obscures much of the pink rectangle because of the very similar reaction times under normal and FES conditions. The two thick curves represent the force trajectories for medium targets; the thin curves represent force trajectories for the high and low targets. The time to target entry after force onset (“rise time”) was substantially longer during FES. (B) Average rise times for each session are plotted against the target height (distance of target above zero force, normalized to the Blocked MVC). Rise time increased with target height under both normal and FES conditions, but the FES times (red symbols) were longer than normal (black symbols) for each monkey (monkey T: circles, monkey A: squares).

Figure 3 indicates that monkey T was able to control the magnitude of brain-controlled stimulation sufficiently well to grade wrist force according to several different target levels. For all sessions in which targets at multiple force levels were presented, the average force differed across targets, even when the targets partially overlapped (1-way ANOVA and Tukey's procedure, p≪0.001). Figure 4A compares average force trajectories for several different targets, aligned with respect to the onset of force under FES (red curves) and normal (black curves) conditions. The thick curves correspond to the medium height flexion target, indicated by the pink and gray rectangles (representing FES and normal conditions, respectively). The thin lines denote forces for the low and high force targets (corresponding targets not shown). Note that here and elsewhere, target *height* refers to the force level corresponding to the bottom of the target, not the difference between top and bottom. The left edge of each target rectangle corresponds to the average time of occurrence of the go tone. Hence, distance from the left edge of a target to time 0 (dashed line) is the average reaction time (RT). The right edge of the target rectangles indicates the end of the average hold time for successful trials.

Overall, the time to success across all targets during FES was 60% greater than normal for monkey T and twice normal for monkey A (t-tests, p≪.001). This difference was potentially the result of several factors, including the monkeys' reaction time, the time to initial target entry, and the stabilization time within the target. In the example in figure 4A, the FES trials had only slightly longer average RT than normal. Across all six sessions for monkey T, the mean RTs were 330 ms and 300 ms for FES and normal conditions, respectively. Although small, the 30 ms difference was significant (t-test, p = 0.006). For the two sessions with monkey A, the difference was larger, with average RTs of 520 ms (FES) and 270 ms (Normal) (t-test, p≪0.001). The force rise time during FES (measured from the onset of force to initial target entry) was also longer than normal for both monkeys (t-tests, p≪0.001). This difference was target dependent, and is summarized in figure 4B as a function of the target height. The difference varied from roughly 100 ms for the lowest targets to 200 ms for the highest. The regression lines on panel B are fitted to the combined data from both monkeys in each condition.

**Figure 5:**
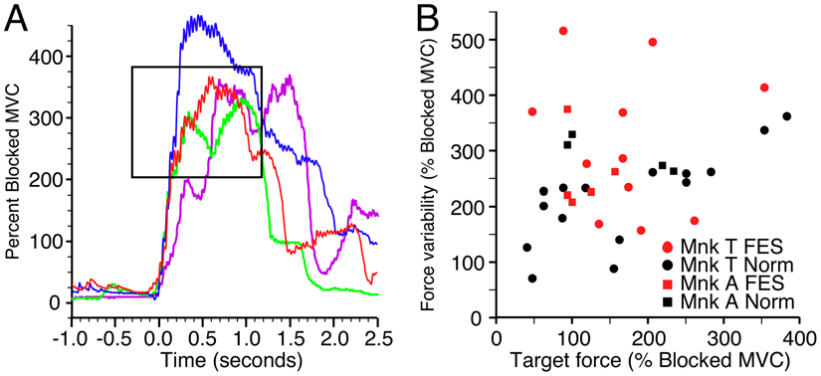
Variability of normal and FES-generated force. (A) Examples of FES trials for monkey T include some that remained within the target from the time of entry until success (red and green traces), and some that undershot (purple trace) or overshot (blue trace) the target. We quantified the variability of the force by calculating its standard deviation (SD) during the period between time of initial target entry and time of successful trial completion. (B) SD is shown as a function of target height under FES (red) and normal (black) conditions. Variability was greater during FES than during normal trials for both monkeys (monkey T: circles, monkey A: squares).

Finally, the monkey's ability to enter the target without subsequently over- or under-shooting was also an important determinant of the time required to achieve a successful trial. Figure 5A illustrates the time course of a number of representative trials from monkey T. The red and green traces entered and stayed within the target for more than the required 500 ms. In contrast, the blue trace rapidly overshot the target before stabilizing within it. Finally, the purple trace undershot the target before being corrected. The high frequency tremor evident in these individual trials was due to incompletely fused muscle contractions. These examples demonstrate that the monkey was able to achieve different force levels voluntarily, and to detect and modify incorrect force levels relatively quickly, although the variability of the FES force was somewhat greater than normal. We quantified this variability by calculating the standard deviation of the force between the time of initial target entry and the end of the trial (Figure 5B). The variability for FES trials was approximately 50% higher than normal for both monkeys, and added 240 ms to the trial length for monkey T (t-test, p≪0.001) and 60 ms for monkey A (t-test, p = 0.2).

**Figure 6:**
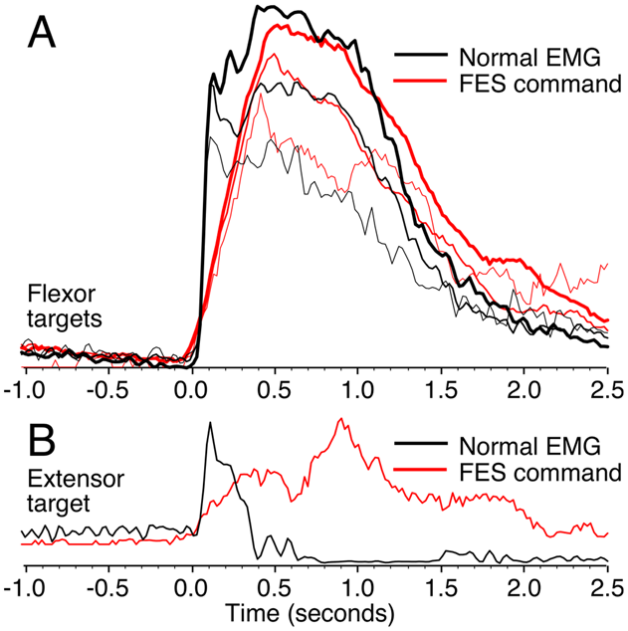
Comparison of EMG during normal conditions and stimulator commands during FES conditions. (A) Average signals corresponding to the three flexion targets are indicated by curves of different thickness. EMG (black) and stimulator pulse-width commands (red) were aligned to the onset of the corresponding force signal and averaged across trials. Although the rise time of the stimulator command was somewhat longer than that of the normal EMG, the correspondence between the shapes of these signals is otherwise quite striking. (B) Same comparison for the extension target. The flexor muscles were typically activated weakly during extension under both normal (black) and FES (red) conditions, although with somewhat different time courses.

Beyond a comparison of the monkeys' normal behavior with that under FES, we sought to compare the real-time stimulator commands to the normal pattern of EMG to determine how well the brain interface replicated natural control (Figure 6). As in figure 4, all traces have been aligned to the onset of force. The black traces represent the EMG signals recorded from FCU under normal conditions. Red traces show the stimulator commands used to activate FCU during an FES session for the same set of targets. The three different flexor target conditions are illustrated in panel A by lines of different thickness. Overall, there was a close similarity in the shapes of the EMG and FES curves: both scaled in a very similar fashion with target height. There was, however, a difference of 200–300 ms in the rise time for the larger targets, which accounts for the difference in force rise times in figure 4.

Although the nerve block did not affect extensor muscles, targets requiring extension force were occasionally included in FES sessions (monkey T: 6 sessions; monkey A: 1 session). During normal, unblocked conditions, there was a low level of cocontraction of flexor and extensor muscles at the beginning of extension trials. The black curve in Figure 6B illustrates such activity in FCU. Likewise, low levels of flexor muscle stimulation often occurred during extension trials (see the single extension trial in Figure 3 and Figure 6B, red curve). During FES flexion trials, stimulation typically preceded the onset of force. However, during extension trials, the monkeys typically began to generate normal extension force somewhat before the onset of flexor muscle stimulation, perhaps in part because of the relatively slow rise-time of FES generated force described above.

It is important to note that the electrical stimulation of muscles activates only a subset of fibers in each muscle, that the recruitment order of these fibers is approximately reversed from normal, and that there is no rate modulation component at all. Given these differences between normal and FES activation of muscles, we consider the overall similarity of the command signal to normal flexor EMG to be remarkable.

## Discussion

We have demonstrated neurally activated FES that provided two monkeys with the ability to exert continuous voluntary, control over the wrist flexor musculature despite temporary paralysis. This was accomplished by using the activity of an ensemble of motor cortex neurons to control the simultaneous, stimulation-driven contraction of four paralyzed muscles. There is no obvious reason why these essential results cannot be scaled up to significantly larger numbers of muscles to allow control of more complex, dexterous movements. These experiments serve as a proof of concept that a similar neuroprosthesis might restore the voluntary control of basic hand movements to a spinal cord injured human patient. Some of the preliminary results for the first monkey (monkey A), dealing with the development of the nerve block and FES methods, have been previously reported [28].

In general, monkey T achieved greater gains in both strength and precision of control through FES than did monkey A. This was probably due in large part to our use of percutaneous muscle electrodes in monkey A, which were less stable and typically less effective in generating force than were the chronically implanted intramuscular electrodes used for monkey T. In addition, the need to rely on percutaneous lidocaine injections rather than the implanted cannula system for peripheral nerve blocks resulted in fewer, shorter duration experiments with monkey A. Not only did this affect our ability to refine our methods, it also limited the amount of time that monkey A was able to adapt to the system. Finally, we were typically able to record about 20% more neurons from monkey T than monkey A, which may also have had some effect. Despite these differences, we were pleased with how quickly both monkeys learned to control the FES system, typically making the transition from the blocked state to FES control in a matter of minutes. This was presumably the consequence of our having mapped the neural discharge onto muscle activity sufficiently closely to that of the natural pattern such that extensive learning was not required to perform the basic task.

Other studies have explored the possibility of brain controlled FES. Limited wrist force generation was recently achieved by a system that used the discharge of 1 or 2 neurons to directly control the activity of 1 or 2 wrist muscles [29]. Control was only possible when the monkey learned to suitably modulate the activity of the individual control neurons. In an earlier study, a human Freehand neuroprosthesis user was able to trigger pre-programmed hand opening and closing movements through EEG recordings of frontal lobe beta band activity [6]. However, subsequent reviews suggested that the signal was at least partially contaminated by EMG [30]. Another study described the use of bursts of beta band EEG activity from imagined foot movement to trigger pre-programmed transcutaneous FES for grasp in a human patient [31]. More recently, the same group used the power in several EEG frequency bands to control transitions between grasp phases in a patient implanted with a Freehand prosthesis [7]. In each of these EEG studies, significant training was required to master the control, despite its pre-programmed nature. In contrast, in our experiments there appeared to be relatively little training needed.

Several groups have used neural activity recorded from electrodes implanted in the cortex to predict the position of a monkey's limb during normal movement [9], [10], [32], [33]. The accuracy of these predictions can be evaluated by comparison with the actual kinematics of the movements. Reported accuracy has been quite similar to the accuracy of our EMG predictions [27]. In several cases, real-time predictions of limb kinematics have been used to allow both monkeys and humans to learn to control a computer cursor or robotic limbs with one, two, or three degrees of freedom [12], [13], [14], [15]. Movement time under such conditions has typically been 2 to 3 times longer than normal. The trial times and force stability of our brain-controlled, FES-induced movements compare quite favorably to these results. However, note that although the control signals for the four muscles in our experiment were independently generated, the behavioral task required the monkeys to control only a single degree of freedom. Human hand FES grasps are typically controlled by a single degree of freedom command signal, and are also slower than normal. Still, the many differences between the FES grasps currently in clinical use and our simple wrist task prevent any meaningful quantitative comparisons between our results and current grasp FES systems.

We are working to increase the force that can be produced by stimulation, as well as the number of controlled muscles. We anticipate that it will be possible to produce voluntarily controlled grasp movements using these brain-controlled FES methods. It is worth noting that the multi-electrode array used here to record neural activity from the monkeys' brains has been implanted in a small number of human patients [12]. We consider the prospect that this new technology could be used to restore more natural hand and arm movements to spinal cord injured patients to be quite exciting.

## Materials and Methods

### Ethics Statement

All animal care, surgical, and research procedures are consistent with the Guide for the Care and Use of Laboratory Animals and were approved by the Institutional Animal Care and Use Committee of Northwestern University. Nonhuman primates are an important experimental model in the investigation of motor control. The motor areas of the central nervous system as well as the musculoskeletal system are very similar to those of humans. Macaque monkeys are not endangered, and are in common use in many different laboratories studying motor control, which allows the efficient comparison of related experiments. There is currently no alternative method to record the activity of single cortical neurons during behavior. We take great care that these animals are comfortable and remain in good health, both because of the potential humane considerations, and because an animal in ill health is unlikely to cooperate as well as one that is healthy.

### Intramuscular Electrodes and Nerve Blocks

Both monkeys had a 100-electrode array (Blackrock Microsystems) chronically implanted in the hand area of motor cortex. The surgical details have been previously described [27]. Neural data were collected using a 96-channel acquisition processor system (Plexon, Inc.). Intramuscular electrodes for bipolar recording and monopolar stimulation were either inserted percutaneously for several weeks (monkey A) or implanted chronically (monkey T).

Peripheral nerve blocks were achieved with percutaneous injection of Lidocaine or Bupivacaine in combination with epinephrine directly to the median and ulnar nerves (monkey A) or via chronically implanted nerve cuffs and injection cannulae (monkey T). By blocking the median and ulnar nerves proximal to the elbow, the flexor muscles of the wrist and fingers and the intrinsic hand muscles were all paralyzed. Nerve blocks were checked periodically for an absence of EMG and sensation, and by the evaluation of dexterity and measurement of maximum wrist strength. Essentially normal muscle strength returned within 3–4 hours of the initial injection of Lidocaine.

### Experimental Task

The monkeys viewed a cursor that was controlled by isometric wrist force and displayed on a video monitor. During six experimental sessions, Monkey T was given randomly intermixed force targets that included a single extension target and 1–3 flexion targets. In two experimental FES sessions, Monkey A was given blocks of trials, each consisting of a single flexion target, occasionally with an additional extension target. The monkeys had 2–3 seconds (4–5 seconds under FES conditions) to move the cursor to a target and to hold it there for 500 ms, for a liquid reward.

Estimates of maximum voluntary contraction (MVC) were made under normal conditions prior to nerve blocks (nine experiments for monkey T, four for monkey A), after the nerve blocks were fully in effect, and then again under blocked conditions when the monkeys were using brain controlled muscle stimulation. In each condition, MVC was estimated by averaging peak force as the monkeys were encouraged to match increasingly high targets for several minutes. The five highest force peaks were averaged to determine the MVC. Both monkeys learned to generate some force with unblocked proximal muscles that tended to increase the Blocked MVC, such that the strength increase provided by FES was probably somewhat underestimated (supplementary materials, “Methods S1”).

### EMG Decoding

EMG signals were sampled at 2000 or 2500 Hz, rectified, filtered, and downsampled to 50 Hz. EMG signals were then predicted using 25 of the available 80 or more neural signals as inputs. These 25 signals were chosen based on the predictive capability of each individual neural signal [34], as well as on the unique character of this capability relative to that of the other recorded neurons [35]. Consideration was also given to the stability of the action potential waveforms over preceding experimental sessions. We calculated multiple-input impulse responses between the neural signals and each of the recorded muscles using a Weiner cascade model (a dynamic linear system followed by a static nonlinearity) [36]. Each impulse response was a causal linear filter of length 0.5 seconds. Thus the output of the real-time system was a weighted, linear combination of the recent history of 25 neural signals, transformed by a 2^nd^ or 3^rd^ order polynomial that implemented the static nonlinearity. The effect of the nonlinearity was to introduce a threshold that eliminated low levels of noise in the predictions, and to increase the gain of the transfer function for the prediction of peak EMG activity (see supplementary materials, “Methods S1”, for more detail).

### Functional Electrical Stimulation

A computer-controlled stimulator (Crishtronics, Cleveland, OH) delivered monopolar, charge-balanced stimuli using a single common return electrode placed on the skin over the elbow. During each experiment, a single electrode was stimulated in each of four muscles: palmaris longus, flexor digitorum sublimis, flexor carpi ulnaris and either flexor digitorum profundus or flexor carpi radialis. In keeping with standard practice in FES applications, variation in stimulation pulse width was used to grade muscle contraction [37], [38]. For any given muscle, pulse width ranged from a threshold width necessary to generate measurable force to a maximum of 200 µs. Stimulus frequency was set to 25 Hz in order to achieve nearly completely fused contractions. The current was fixed for each electrode (typically 8–12 mA). The EMG predictions (described above) were further scaled and thresholded to produce the corresponding stimulus pulse-width commands. The scaling and thresholding parameters for each muscle were initially estimated from the statistics of the EMG predictions and the characteristic force produced by fixed stimulus trains. They were typically further refined at the beginning of each FES session to maximize FES MVC and to minimize undesired low level stimulation between trials. Once established, these parameters were fixed for the duration of a session.

## Supporting Information

Methods S1 This file contains a more extensive description of the methods.
(0.04 MB DOC)

Brain Controlled FES S1 This is a video taken of the screen, showing the required force target, and the cursor that is being controlled by the monkey through the brain-controlled FES system. “Interface off” indicates a catch trial during which the input to the stimulators has been turned off, in order to test the monkey's ability to do the task without FES assistance.
(1.55 MB MOV)
