# Supplementary material for: Toward the Restoration of Hand Use to a Paralyzed Monkey: Brain-Controlled Functional Electrical Stimulation of Forearm Muscles
Source: PLoS One. 2009 Jun 15;4(6):e5924. doi: 10.1371/journal.pone.0005924 (PMC2691481; doi:10.1371/journal.pone.0005924)
Supplement: Methods S1 — This file contains a more extensive description of the methods. (0.04 MB DOC) [file pone.0005924.s001.doc]

**Materials and Methods**

Surgical implantation of intramuscular electrodes and nerve cuffs

Pairs of stranded, stainless steel leads (Cooner 632) were inserted into each muscle for recording. An individual lead from each pair was used for monopolar stimulation. For monkey A, the leads were inserted percutaneously under ketamine-xylazine anesthesia, taped to the skin, and routed to a connector using the method described by Park and colleagues [1]. For monkey T, the electrodes were chronically implanted under Isoflurane anesthesia, and the leads were routed subcutaneously to a back connector [2]. In the same procedure, custom-made nerve cuffs were implanted around the median and ulnar nerves at sites just proximal to the elbow and the origin of the median nerve branches to flexor carpi radialis and pronator teres. The cuffs were connected via cannulae to subdermal injection ports (Mentor Injection Domes) implanted on the upper arm. Antibiotics and analgesics were administered post-operatively.

Nerve Blocks

Local anesthetic (2 or 3.3% Lidocaine or 0.75% Bupivacaine) was used to block the median and ulnar nerves. Epinephrine was essential for successful motor blocks (1:100,000 for lidocaine, 1:200,000 for bupivacaine). For monkey T, the agent was simply injected into the subdermal injection ports. Monkey A, having no cuffs, was lightly anesthetized with Isoflurane and the nerve blocks were implemented by percutaneous injections directly to the nerves. This involved locating each nerve using stimulation (0.3-0.5 mA, 100 µsec pulse widths) through a B. Braun Stimuplex needle, which was then used to inject the anesthetic. Experiments were not started until monkey A had recovered from the gas anesthesia.

By blocking the median and ulnar nerves, the flexor muscles of the wrist and fingers, and the intrinsic hand musculature were all paralyzed. Nerve blocks were checked periodically throughout each FES session by the absence of EMG and sensation, evaluation of finger dexterity as the monkey attempted to grasp small food items, and measurement of maximum wrist strength.

Maximum Voluntary Contraction (MVC)

Measuring MVC in these experiments was more difficult than in experiments with cooperative human subjects. It is important to realize the limitations of our MVC measurement, and the fact that it deemphasized transient force peaks. To estimate MVC, the monkeys were encouraged to match increasingly high force targets during a several minute test period. In some cases, an automated search routine was used that differentially rewarded higher targets. Force was averaged in a 0.5 second window around each peak, and the five highest force peaks were averaged to determine the MVC. Normal MVC was estimated on the day before the FES experiments for monkey A, and just prior to the nerve block in each FES session for monkey T.

Both monkeys learned to “cheat” in various ways that resulted in small measured forces that did not appear to be only the result of the contraction of wrist musculature. These included elbow flexion, extension and forearm supination movements, as well as inertial forces generated at the wrist by swinging the upper arm. These effects were primarily noticeable in the blocked state without FES, when the monkey was struggling to complete the task. They did not seem to be employed when cortical FES was available. As a result, they appeared to increase Blocked MVC, without affecting the FES MVC, such that the apparent strength increase provided by FES may have been somewhat underestimated.

EMG Decoding

EMG signals were predicted using a Weiner cascade model: a dynamic linear system followed by a static nonlinearity [3]. The EMG activity was predicted using a linear system with multiple inputs and a single output, in which each neural input was convolved with its finite impulse response function *h_k_*. As a result, the system output was simply a result of a weighted, linear combination of *N* inputs stretching from the present (lag 0) to *M* points in the past. We used a causal linear filter of length 500 ms. Estimation of *h_k_* was computed using the auto- and cross-correlation matrices of the system inputs and outputs in a designated block of training data [4,5]. In the real-time implementation reported here, we reduced sampling rates from 100 to 50 Hz and used a standard matrix inversion rather than the SVD pseudo-inverse described previously[6].

In order to reduce model complexity and the likelihood of overfitting the data, we used several different criteria to identify a subset of input neurons. Primarily, we considered the amount of unique information contributed by each signal to the EMG predictions using a QR ranking technique [7]. Essentially, this is an iterative method that identifies the unique contribution of each neural input signal to the prediction of a particular output after taking into account the information available from all other potential inputs. The neural signal that contributes the least unique information is dropped from the pool of potential inputs. The process is then repeated with the remaining neural signals. The iterative process is necessary since the unique contributions of the remaining signals change, as each successive signal is dropped. Neural signals were chosen as decoder inputs on the basis of their ranks once all the signals had been so scored. Neurons were then occasionally rejected from this set if, 1) the neuron had not been recorded stably for several preceding days, or 2) if, compared to all other neurons, a given neuron was highly uninformative for all muscles. By this means, we selected 25 of the available (typically 80 or more) neural signals to be used as inputs.

The training data used to create the models included both wrist flexion and extension targets. The targets were typically less than 30% of normal MVC and were similar, but rarely identical, to the FES targets. Ten minutes of training data (roughly 120 trials) were used for monkey A, and 20 minutes (over 250 trials) for monkey T. Neuron discrimination settings were established during the training data session, and no effort was made to adjust the sorting parameters for the subsequent FES session. However, significant shape changes in waveforms between these sessions were relatively infrequent.

References

1. Park MC, Belhaj-Saïf A, Cheney PD (2000) Chronic recording of EMG activity from large numbers of forelimb muscles in awake macaque monkeys. J Neurosci Methods 96: 153-160.

2. Miller LE, van Kan PLE, Sinkjaer T, Andersen T, Harris GD, et al. (1993) Correlation of primate red nucleus discharge with muscle activity during free-form arm movements. J Physiol London 469: 213-243.

3. Hunter IW, Korenberg MJ (1986) The identification of nonlinear biological systems: Wiener and Hammerstein cascade models. Biol Cybern 55: 135-144.

4. Hunter IWKRE (1983) Two-sided linear filter identification. Med & Biol Eng& Comput 21: 203-209.

5. Perreault EJ, Kirsch RF, Acosta AM (1999) Multiple-input, multiple-output system identification for characterization of limb stiffness dynamics. Biol Cybern 80: 327-337.

6. Pohlmeyer EA, Solla SA, Perreault EJ, Miller LE (2007) Prediction of upper limb muscle activity from motor cortical discharge during reaching. Journal of Neural Engineering 4: 369-379.

7. Westwick DT, Pohlmeyer EA, Solla SA, Miller LE, Perreault EJ (2006) Identification of Multiple-Input Systems with Highly Coupled Inputs: Application to EMG Prediction from Multiple Intracortical Electrodes. Neural Comput 18: 329-355.
